# Association Between Education Levels and Sedentary Behavior With Depression Among US Adults

**DOI:** 10.1002/brb3.70615

**Published:** 2025-06-10

**Authors:** Chun Yang, Tiankuo Gao, Yichen Zhang, Cuicui Feng, Kai Zhang

**Affiliations:** ^1^ Beijing Anzhen hospital, Capital Medical University Beijing China; ^2^ Longyan University Longyan China

**Keywords:** depression, education level, NHANES, sedentary behavior

## Abstract

**Background:**

Earlier studies have proposed the effect of education level and sedentary behavior (SB) on the incidence of depression in adults. However, the association between the combination of education level and SB and depression in adults has not yet been investigated.

**Methods:**

This study population consisted of US adults (aged ≥18 years) who participated in the National Health and Nutrition Examination Survey (NHANES) from 2007 to 2018. A multivariable logistic regression model was employed to assess the association between education level, SB, and depression.

**Results:**

Of the 29,822 participants (weighted mean [SE] age, 47.9[0.2] years; 51.2% male) in our study cohort. Depression was negatively linked to the higher education level (adjusted OR = 0.68 [95% CI: 0.57–0.81], Model 4) and positively correlated to higher SB (adjusted OR = 1.58 [95% CI: 1.34–1.87]). The ORs [95% CIs] for depression were 1.40 [1.13–1.74], 1.68 [1.20–2.35], and 1.78 [1.42–2.22], respectively, among lower education groups sitting < 6 h a day (h/d), 6–8 h/d and ≥ 8 h/d compared with higher education/ sitting < 6 h/d groups (Model 3). Among participants with higher education, those who engaged in SB ≥ 8 h/d had a 1.53‐fold [95% CI, 1.31–1.79] increased risk of depression compared with those who sat for <6 h/d (*p* < 0.0001; Model 4).

**Conclusions:**

A lower education level and prolonged SB are independently and jointly associated with an increased risk of depression. Interventions that aim to reduce SB, especially among those with lower educational levels and also among those with higher educational levels who sit for more than 8 h per day, may help reduce the prevalence of depression.

## Graphical abstract

Higher education is associated with lower depression risk, but excessive sedentary behavior increases that risk regardless of education level. Our study reveals a joint association of low education and prolonged sitting time with the highest depression prevalence among US adults.

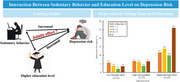

## 1 Introduction

Depression is a widespread and incapacitating mental health disorder affecting over 280 million individuals worldwide. It is a leading cause of disability and contributes to the global disease burden (Ferrari et al. 2024; GBD 2019 Mental Disorders Collaborators 2022). The condition imposes profound personal, social, and economic costs, with direct impacts on productivity and healthcare systems (Smith 2014; Ferrari et al. 2013; Sartorius 2001). Although the etiology of depression is multifactorial, adjustable elements like societal factors, notably educational level, and daily habits, including the amount of time spent sedentary, play a crucial role in shaping one's vulnerability to depression (Cohen and Syme 2013; Esch et al. 2014; McFarland and Wagner 2015; Lorant et al. 2003; Covenant et al. 2024; Yang et al. 2024; Feng et al. 2024).

Higher educational attainment is generally associated with improved health outcomes due to better socioeconomic conditions, enhanced health literacy, and greater access to healthcare resources (Esch et al. 2014; Aartsen et al. 2017). Education further cultivates mental fortitude and flexible coping strategies, which may reduce the likelihood of experiencing mental health issues, such as depression (Cohen et al. 2020; Mirowsky 2017). While sedentary behavior (SB), any waking activity that expends 1.5 metabolic equivalents while seated, reclined, or lying down, is on the rise (Yang et al. 2019) and correlates with various adverse health effects, depression being one of them (Zhai et al. 2015; Hallgren et al. 2020).

However, paradoxically, highly educated individuals are more likely to occupy sedentary occupational roles that demand prolonged cognitive engagement (e.g., office‐based professions), which may inadvertently elevate depression risk despite their socioeconomic advantages (Kantomaa et al. 2016). While prolonged occupational sitting has been associated with adverse cardiometabolic outcomes and poor self‐rated health (Gilson et al. 2011), its specific links to depression remain underexplored. Notably, emerging evidence suggests that the mental health impacts of SB may depend on its cognitive context: passive SB (e.g., television viewing) is consistently linked to higher depression risk, whereas mentally active SB (e.g., reading, cognitively demanding work) may confer neutral or even protective effects (Hallgren et al. 2020; Huang et al. 2020). This distinction raises a critical question—do the cognitively engaging tasks inherent to high‐education professions mitigate the psychological harms of occupational SB, or does the prolonged sitting override the mental health benefits of education? Current studies have largely examined SB, education, and depression in isolation (McFarland and Wagner 2015; Yang et al. 2024; Cohen et al. 2020; Hallgren et al. 2020; Huang et al. 2020; Wang and Peiper 2022; Sanchez‐Villegas et al. 2008; Jiang et al. 2024; Huang et al. 2021; Daneshmandi et al. 2017; Teychenne et al. 2010), leaving their synergistic effects poorly understood. Clarifying this interaction is essential to determine whether interventions should target SB reduction universally or prioritize cognitive engagement strategies tailored to educational strata.

This research fills the knowledge gaps by utilizing information from the National Health and Nutrition Examination Survey (NHANES) to investigate the independent and combined effects of educational attainment and SB on depression risk. Through clarifying these interactions, it seeks to provide a foundation for developing data‐driven approaches for reducing sedentary time and mitigating depression risk across different educational groups.

## 2 Methods

### 2.1 Study Design and Participants

The research utilized information gathered through NHANES, a comprehensive and nationally representative study managed by the National Center for Health Statistics (NCHS). To guarantee robust statistical analysis and inclusion of critical factors associated with education, SB, and depressive symptoms, the study compiled data across several survey cycles.

**FIGURE 1:**
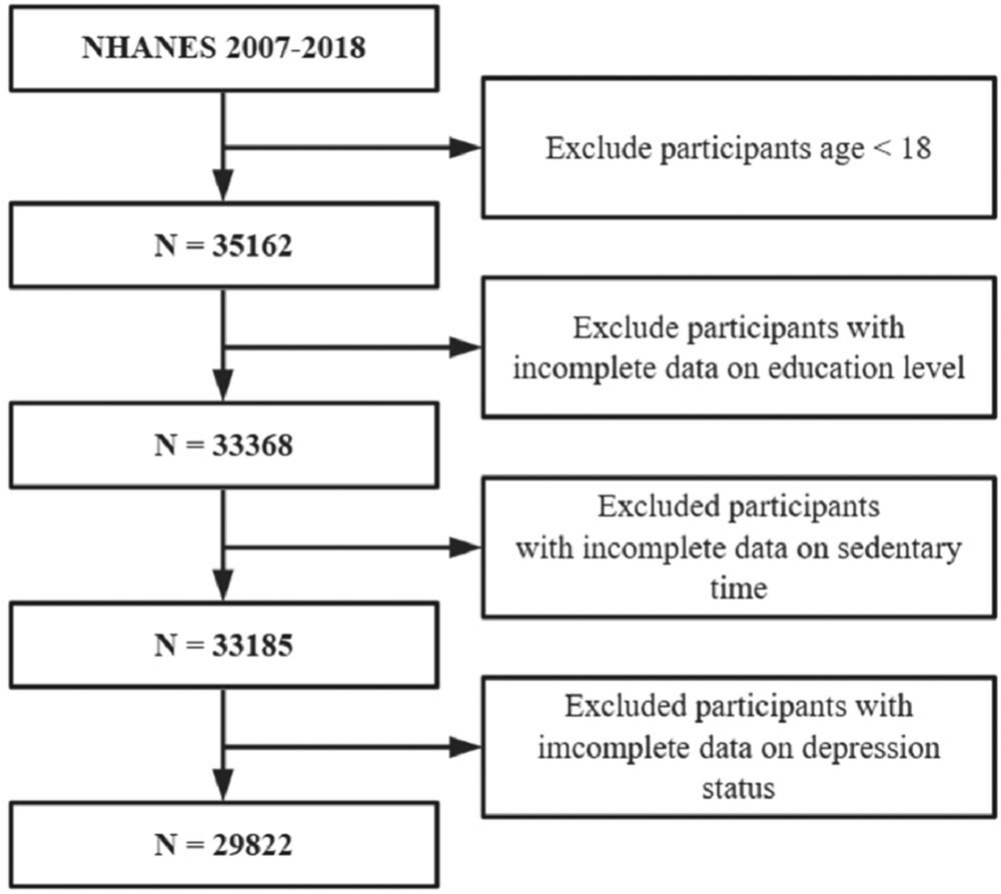
Flowchart.

The research focused on adults, 18 years and older, who had completed the NHANES questionnaires and assessments relevant to the analysis. Participants with incomplete data on education level, sedentary time, or depression status were excluded (Figure 1). Following the application of exclusion criteria, the study encompassed a sample of 29,822 participants for analysis. The intricate, multistage sampling methodology of the survey was factored into every statistical evaluation to guarantee that the findings reflect the broader national population.

### 2.2 Variables

The study utilized the Patient Health Questionnaire‐9 (PHQ‐9), comprising nine items with a scoring system from 0 to 3 per item, to evaluate depressive symptoms experienced in the two weeks preceding the survey (Kroenke et al. 2010). The reliability of the PHQ‐9 was assessed using SPSS 26.0, yielding a Cronbach alpha coefficient of 0.84 (Kroenke et al. 2010). The total score on the PHQ‐9 ranged from a minimum of 0 to a maximum of 27 points (Kroenke et al. 2010). Depressive symptoms were classified as ≥10 points (Kroenke et al. 2001). Consequently, depressive symptoms were assigned the following codes: A “0” denoted the absence of symptoms, corresponding to a score between 0 and 9, whereas a “1” signified the presence of symptoms, associated with a score between 10 and 27.

Data on participants' educational levels (the independent variable) was collected through a questionnaire asking, “What is the highest grade or level of school you have completed or the highest degree have received?” The response categories for this question were as follows: “< high school” for those with less than a high school diploma; “high school” for those who had completed high school or obtained a GED (General Education Development or Diploma); and “> high school” for individuals with some college education or an Associate of Arts degree or higher. Participants who chose the options “Refused,” “Don't Know,” or “Missing” were not included in this research analysis. To further assess the robustness of the results, we conducted a sensitivity analysis by stratifying the sample based on education level.

Participants reported their SB using the Global Physical Activity Questionnaire (GPAQ), a tool developed by the World Health Organization (WHO) and incorporated into the NHANES (Armstrong and Bull 2006). The measure of SB was derived from participants' reports of their typical daily sitting time (PAD 680), which encompassed activities like reading, watching TV, computer use, and traveling by car or bus. Referring to a previous study, the time spent engaged in SB was classified into four categories: less than 4 h/day, 4 to 6 h/day, 6 to 8 h/day, and 8 h or more per day (Huang et al. 2021).

Covariates considered in the analysis included age, sex, ethnicity, body mass index (BMI), diet score, sleep score, smoking status, and alcohol consumption. Self‐reported medical diagnoses of hypertension, diabetes mellitus (DM), tumors, atherosclerotic cardiovascular disease (ASCVD), and chronic kidney disease (CKD), were also obtained at baseline. The main analysis incorporated eight social factors: employment status, the ratio of family income to the poverty line (determined by family size), food security (assessed through ten inquiries regarding food scarcity, with full security indicated by no positive responses), educational attainment, access to healthcare (defined by the presence of a regular healthcare facility for non‐emergency consultations), insurance type (private, public, or none), housing status, and marital or cohabitation status.

### 2.3 Statistical Analysis

Adhering to NHANES data usage guidelines, the study considered the intricate aspects of the survey design. The recommended survey weights were applied for the analysis. Continuous data were presented as averages with standard deviations and were analyzed using one‐way ANOVA. For categorical data, weighted counts and proportions were computed using the card method tests (Liu et al. 2020). To enhance comparability, a series of multivariable regression models were constructed: Model 1 (unadjusted); Model 2 (adjusted for age, sex, and ethnicity); Model 3 (further adjusted for age, sex, ethnicity, BMI, dietary score, sleep quality, smoking, alcohol use, hypertension, DM, cancer, CKD, and ASCVD); and Model 4 (including all the above plus social health determinants such as employment, economic status, food security, education, healthcare access, insurance type, home ownership, and relationship status, along with sedentary time and educational level).

## 3 Results

**FIGURE 2:**
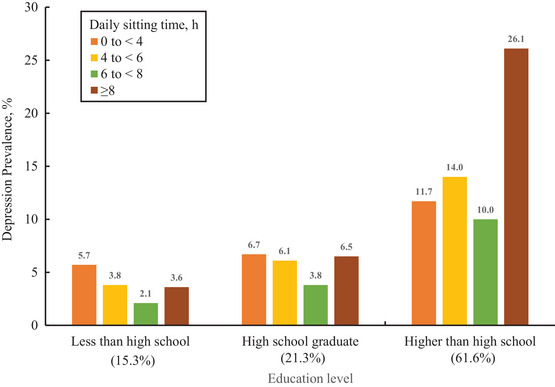
Prevalence of depression by daily sitting time and education level.

Of the 29,822 participants (weighted mean [SE] age, 47.9 [0.2] years; 48.8% male) in the study. Individuals who spent ≥8 h per day sitting were observed to be older (mean [SE] age: 48.2 [0.3] years) compared to those who sat <4 h per day (h/d) (45.8 [0.3] years, Table 1). The ≥8 h/d SB group also had the highest BMI (29.9) and lowest physical activity scores (65.2; all *p* < 0.001). Participants in the sitting <4 h/d group consistently had the lowest prevalence of chronic conditions, including hypertension (45.0%), DM (12.4%), cancer (7.6%), and ASCVD (6.2%), suggesting that less sedentary time correlates with better overall health (all *p* < 0.001, Table 1). Participants who sat ≥8 h per day had higher socioeconomic advantages, including the highest employment rates (81.4%), family income‐to‐poverty ratio (58.9%), access to health care (86.5%), food security (88.6%), and private health insurance (69.2%; all *p* < 0.001, Table 1). A majority, 61.6%, of the study's subjects possessed an education level beyond high school (Figure 2). Of significance, 26.1% of the respondents indicated that they had surpassed a high school diploma and also engaged in extended periods of sitting, exceeding 8 h daily (Figure 2).

**TABLE 1:** Characteristics of the study population by sitting time.

Variable	Total	Sitting time				*p* value
		<4 h/d	4 to <6 h/d	6 to <8 h/d	≥8 h/d	
**Age, years**	47.9(0.2)	45.8 (0.3)	48.4(0.4)	49.5(0.4)	48.2(0.3)	< 0.001
**Gender**						0.946
Female	15150(51.2)	4283(51.0)	3544(51.6)	2328(51.2)	4995(51.2)	
Male	14672(48.8)	4057(49.0)	3557(48.4)	2303(48.8)	4755(48.8)	
**Race**						< 0.001
Mexican‐American	4477(8.5)	2020(15.1)	1029(8.4)	545(6.8)	883(4.9)	
NH Black	6375(11.0)	1586(11.3)	1533(11.1)	1013(10.8)	2243(10.9)	
NH White	12409(67.0)	2738(57.2)	2985(66.9)	2136(70.7)	4550(72.0)	
Other Hispanic	3101(5.8)	1217(9.1)	749(5.9)	383(4.3)	752(4.1)	
Other Race	3460(7.7)	779(7.3)	805(7.7)	554(7.3)	1322(8.1)	
**BMI, kg/m^2^**	29.2(0.1)	28.3 (0.1)	28.7(0.1)	29.4(0.2)	29.9(0.1)	< 0.001
**Smoke status**						< 0.001
Never	16490(55.5)	4789(55.0)	3836(54.4)	2462(54.5)	5403(57.0)	
Former	7236(24.9)	1794(22.6)	1734(24.4)	1210(26.5)	2498(26.1)	
Now	6081(19.6)	1754(22.4)	1526(21.3)	955(19.1)	1846(16.8)	
**Drinking**	24560(86.1)	6552(86.2)	5907(89.3)	3874(89.8)	8227(90.2)	< 0.001
**Sitting Time, hour/day**	6.2(0.0)	2.3(0.0)	4.4(0.0)	6.3(0.0)	10.0(0.0)	< 0.001
**Education level**						< 0.001
Less than high school	7134(15.3)	2892(23.8)	1732(16.0)	941(13.5)	1569(10.1)	
High school graduate	6852(23.1)	2072(27.8)	1771(25.5)	1075(24.1)	1934(17.9)	
Higher than high school	15836(61.6)	3376(48.4)	3598(58.4)	2615(62.5)	6247(72.0)	
**Physical activity score**	73.1(0.5)	79.8 (0.8)	77.9(0.7)	74.2(0.8)	65.2(0.9)	< 0.001
**HEI‐2015**	39.4(0.5)	39.5(0.7)	39.3(0.7)	39.1(0.7)	39.6(0.5)	0.871
**Sleep score**	83.5(0.3)	82.1(0.4)	83.6(0.4)	83.7(0.5)	84.1(0.4)	< 0.001
**Employment**						< 0.001
Not employed	7488(21.3)	2222(23.5)	1797(22.5)	1204(22.1)	2265(18.6)	
Employed, student, retired	22316(78.7)	6110(76.5)	5299(77.5)	3423(77.9)	7484(81.4)	
**Family income to poverty ratio**						< 0.001
<300%	17288(46.6)	5494(62.6)	4262(52.4)	2653(50.2)	4879(41.1)	
≥300%	9850(46.1)	1900(37.4)	2230(47.6)	1619(49.8)	4101(58.9)	
**Food security**						< 0.001
Marginal, low, or very low security	5540(14.2)	1913(18.8)	1349(14.6)	773(13.0)	1505(11.4)	
Full security	24282(85.8)	6427(81.2)	5752(85.4)	3858(87.0)	8245(88.6)	
**Regular health care access**						< 0.001
None or emergency room	5356(16.8)	1960(22.0)	1304(17.4)	723(15.4)	1369(13.5)	
≥One regular health care facility	24464(83.2)	6379(78.0)	5797(82.6)	3908(84.6)	8380(86.5)	
**Type of health insurance**						< 0.001
Government or none	14507(38.1)	4838(48.1)	3578(39.6)	2163(37.4)	3928(30.8)	
Private	15315(61.9)	3502(51.9)	3523(60.4)	2468(62.6)	5822(69.2)	
**Home ownership**						< 0.001
Rent home or other arrangement	11386(32.1)	3393(35.9)	2628(31.1)	1704(31.0)	3661(32.0)	
Own home	17947(66.5)	4791(64.1)	4350(68.9)	2859(69.0)	5947(68.0)	
**Marital status**						0.001
Not married nor living with a partner	12164(36.8)	3180(35.7)	2795(34.9)	1966(37.8)	4223(38.4)	
Married or living with a partner	17645(63.2)	5156(64.3)	4302(65.1)	2663(62.2)	5524(61.6)	
**Hypertension**	16252(50.0)	4235(45.0)	3933(51.3)	2666(53.0)	5418(51.1)	< 0.001
**ASCVD**	3009(8.1)	629(6.2)	704(7.7)	553(9.4)	1123(8.9)	< 0.001
**Diabetes mellitus**	5811(14.6)	1506(12.4)	1351(13.7)	934(15.8)	2020(16.2)	< 0.001
**Cancer**	2952(10.6)	588(7.6)	728(10.9)	570(12.5)	1066(11.6)	< 0.001

Abbreviations: ASCVD, atherosclerotic cardiovascular disease; BMI, body mass index; HEI‐2015, Healthy Eating Index 2015; NH, non‐Hispanic.

Data are presented as mean ± SE or n (%).

There was a notable downward trend in the rates of depression as education levels rose, with the highest incidence noted in individuals who did not complete high school at 13.5%, and the lowest in those who attained an education surpassing high school, at 6.3% (Table 2). Individuals who completed high school experienced a substantial decrease in depression risk, with Model 3 showing an odds ratio (OR) of 0.71 (95% confidence interval (CI) 0.59–0.86, *p* < 0.001) and Model 4 an OR of 0.82 (95% CI 0.69–0.96, *p* = 0.010). This reduction was even more pronounced among those with an education exceeding high school, who had an OR of 0.61 (95% CI 0.51–0.73, *p* < 0.001) in Model 3 and an OR of 0.68 (95% CI 0.57–0.81, *p* < 0.001) in Model 4, when compared to those without a high school diploma (Table 2). The results of the sensitivity analysis are consistent with the result (Table S1).

**TABLE 2:** Odds ratios for the association between education level and depression.

Variable	Event/	Weighted	Weighted	Model 1		Model 2		Model 3		Model 4	
	Participant	Event	Event Percent	OR (95%CI)	*p* value	OR (95% CI)	*p* value	OR (95% CI)	*p* value	OR (95% CI)	*p* value
**Education level**											
<High school	989/7134	4,260,784	13.5	Reference	—	Reference	—	Reference	—	Reference	—
High school	654/6852	4,401,550	9.3	0.65 (0.57,0.75)	<0.001	0.63 (0.54,0.72)	<0.001	0.71 (0.59,0.86)	<0.001	0.82 (0.69,0.96)	0.010
>High school	1108/15,836	7,963,725	6.3	0.43 (0.37,0.49)	<0.001	0.39 (0.34,0.46)	<0.001	0.61 (0.51,0.73)	<0.001	0.68 (0.57,0.81)	<0.001
*P for trend*				<0.001		<0.001		<0.001		<0.001	

*Note*: Model 1, not adjusted. Model 2, adjusted for age, gender, and sex. Model 3, adjusted for age, gender and race, body mass index, diet score, sleep score, smoking status, alcohol consumption, hypertension, diabetes mellitus, cancer, chronic kidney disease, atherosclerotic cardiovascular disease, and educational levels. Model 4, adjusted for age, sex, race, social determinants of health (employment status, family income‐to‐poverty ratio, food security, education level, regular health care access, type of health insurance, home ownership, and marital status) and educational levels.

Abbreviations: CI, confidence interval; OR, odd ratio.

In the stratified analysis by sedentary time, the prevalence of depression increased with sedentary time, climbing from 7.8% (<4 h/d) to 9.0% (≥8 h/d, Table 3). Each additional hour of sitting was linked to an increased risk of depression, with an OR of 1.03 (95% CI: 1.01–1.05] per 1‐h increase in Model 3 (*p* = 0.005) and an OR of 1.07 [95% CI: 1.04‐1.09] per 1‐h increase in Model 4 (*p* < 0.0001; Table 3). A considerable surge in depression risk was exclusively associated with the group that spent 8 or more hours sedentary (Model 3: OR:1.22 [95% CI: 1.03–1.45], *p* = 0.020; Model 4: OR:1.58 [95% CI: 1.34–1.87], *p* < 0.001) compared to those who sat <4 h/d. In contrast, no significant difference in depression prevalence was found between those who sat for 4–6 h or 6–8 h a day and the group sitting for less than 4 h (Table 3).

**TABLE 3:** Odds ratios for the association between sitting time and depression.

Variable	Event/	Weighted	Weighted	Model 1		Model 2		Model 3		Model 4	
	Participant	Event	Event Percent	OR (95%CI)	*p*value	OR (95% CI)	*p* value	OR (95% CI)	*p* value	OR (95% CI)	*p* value
**Sitting time**											
<4 h/d	734/8340	3,867,357	7.8	Reference	—	Reference	—	Reference	—	Reference	—
4 to <6 h/d	610/7101	3,601,644	7.3	0.93 (0.80,1.08)	0.370	0.95 (0.82,1.11)	0.550	1.03 (0.85,1.25)	0.750	1.06 (0.89,1.27)	0.500
6 to <8 h/d	422/4631	2,487,506	7.6	0.97 (0.82,1.15)	0.710	1.00 (0.85,1.19)	0.960	0.95 (0.78,1.16)	0.600	1.14 (0.94,1.39)	0.180
≥8 h/d	985/9750	6,669,553	9.0	1.16 (1.00,1.35)	0.050	1.20 (1.03,1.41)	0.020	1.22 (1.03,1.45)	0.020	1.58 (1.34,1.87)	<0.001
Per 1 h/d increase				1.03 (1.01,1.05)	0.010	1.03 (1.01,1.05)	0.001	1.03 (1.01,1.05)	0.005	1.07 (1.04,1.09)	<0.001
*p for trend*				0.020		0.010		0.020		<0.001	

Model 1: Not adjusted. Model 2: Adjusted for age, gender and sex. Model 3: Adjusted for age, gender and race, body mass index, diet score, sleep score, smoking status, alcohol consumption, hypertension, diabetes mellitus, cancer, chronic kidney disease, atherosclerotic cardiovascular disease, and sitting hour.

Model 4: Adjusted for age, sex, race, social determinants of health (employment status, family income‐to‐poverty ratio, food security, education level, regular health care access, type of health insurance, home ownership, and marital status) and sitting time.

OR, odd ratio; CI, Confidence interval.

Joint analyses revealed an increased risk of depression for lower education and high sedentary time (Table 4). After adjustment for demographic and health‐related covariates (Model 3), the ORs [95% CIs] for depression were 1.40 [1.13–1.74], 1.68 [1.20–2.35], and 1.78 [1.42–2.22], respectively, among lower education groups sitting <6 h/d, 6–8 h/d, and ≥8 h/d compared with higher education groups (sitting <6 h/d, Table 4). The patterns were consistent when social determinants were factored into the analysis (Model 4). Among participants with higher levels of education, those who engaged in SB ≥8 h/d had a 1.53 times [95% CI, 1.31–1.79] higher chance of depression compared with those who sat <6 h per day, after adjustment for demographic and social determinants (*p* < 0.001; Model 4, Table 4). However, no significance was observed after adjustment for demographics alone (Model 2) or demographics and health‐related covariates (Model 3, Table 4). Notably, the higher education/6‐8 h/d SB group did not exhibit a significant link to depression risk across all models (Table 4). The results of the sensitivity analysis are consistent with the primary findings, further confirming the robustness of the analysis. Detailed results are shown in Table S2.

**TABLE 4:** Odds ratios for the association between the combination of education level and sitting time, and depression.

Variable	Event/	Weighted	Weighted	Model 1		Model 2		Model 3		Model 4	
	Participant	Event	Event Percent	OR (95%CI)	*p* value	OR (95% CI)	*p* value	OR (95% CI)	*p* value	OR (95% CI)	*p* value
**High education**											
<6 h/d	785/10817	2,600,772	6.3	Reference	—	Reference	—	Reference	—	Reference	—
6 to <8 h/d	263/3690	1,822,437	6.4	0.98 (0.83,1.17)	0.860	1.00 (0.84,1.20)	0.980	0.86 (0.69,1.06)	0.150	1.05 (0.86,1.28)	0.150
≥8 h/d	714/8181	5,400,965	8.1	1.26 (1.09,1.47)	0.003	1.27 (1.09,1.48)	0.002	1.15 (0.97,1.36)	0.100	1.53 (1.31,1.79)	<0.001
**Low Education**											
<6 h/d	599/4624	1,000,872	12.7	1.93 (1.63,2.28)	<0.001	2.03 (1.70,2.44)	<0.001	1.40 (1.13,1.74)	0.003	1.33 (1.10,1.62)	0.004
6 to <8 h/d	159/941	665,069	15.1	2.55 (2.00,3.24)	<0.001	2.66 (2.09,3.40)	<0.001	1.68 (1.20,2.35)	0.003	1.73 (1.31,2.28)	<0.001
≥8 h/d	271/1569	1,268,589	16.9	2.92 (2.41,3.53)	<0.001	3.15 (2.59,3.82)	<0.001	1.78 (1.42,2.22)	<0.001	1.89 (1.51,2.36)	<0.001

*Note*: Model 1, not adjusted. Model 2, adjusted for age, gender and sex. Model 3, adjusted for age, gender and race, body mass index, diet score, sleep score, smoking status, alcohol consumption, hypertension, diabetes mellitus, cancer, chronic kidney disease and atherosclerotic cardiovascular disease. Model 4, adjusted for age, sex, race and social determinants of health (employment status, family income‐to‐poverty ratio, food security, education level, regular health care access, type of health insurance, home ownership, and marital status).

Abbreviations: CI, confidence interval; OR, odd ratio.

## 4 Discussion

Within the US nationally representative cohort of adults, 26.1% reported having more than a high school education and sitting for over 8 h daily. Prolonged SB (sitting more than 8/d) escalates the risk of depression, regardless of education level. Individuals with higher education also had increased depression risk, irrespective of the amount of sitting time. During joint analyses, the association of prolonged SB with increased depression risk was observed among US adults with lower educational levels, as much as a 1.78‐fold to 1.89‐fold increase in the risk of depression. Our observational data suggest that higher educational attainment was associated with reduced depression risk at moderate sedentary durations (6–8 h/d), whereas sitting time exceeding 8 h/d remained correlated with elevated depression risk regardless of education level. To our knowledge, this is the first study to investigate the joint associations of sitting time and education levels with depression risk in a nationally representative sample of adults.

Given the effects of lifestyles on mental health, sitting‐related SB are important research target. Our study found that depression risk increased in the ≥8 h/d SB group compared to the <4 h/d group. Though no significant differences were noted for those sitting 4–8 h/d, each additional hour of sitting was linked to higher depression risk (Table 3), indicating a dose‐dependent relationship between prolonged sedentary time and elevated depression risk. Consistent with our observations, a multitude of prior research has established a positive correlation between SB and the risk of depression in different populations (Teychenne et al. 2010). Excessive SB (over 42 h per week of screen time) increased the risk by 31% when compared with those spending <10.5 h.wk(‐1) (Sanchez‐Villegas et al. 2008). US high school students using digital devices for 3+ h per school night had 1.61 times [95% CI: 1.41–1.85] higher odds of depressive symptoms (Wang and Peiper 2022). Total SB raises depression risk [OR: 1.49, 95% CI: 1.24–1.79] in older adults (Jiang et al. 2024; Hu et al. 2022).

Meanwhile, numerous evidence demonstrated higher educational attainment correlates with fewer depressive symptoms (Cohen et al. 2020). Individuals with higher education tend to be office workers, where the typical workday involves about 6.29 h of sitting during an 8‐h period, a routine that can lead to a variety of negative health consequences (Daneshmandi et al. 2017). M. T. Kantomaa et al. also reported that the challenges associated with unhealthy behaviors in young adults with advanced educational backgrounds face challenges related to high sedentary time, particularly on weekdays (Kantomaa et al. 2016). Previous findings are not insufficient to figure out whether higher education attainment may moderate the relationship between SB and depression risk. In our study, we found higher educational attainment was associated with reduced depression risk at moderate sedentary durations (6–8 h/d), whereas sitting time exceeding 8 h/d remained correlated with elevated depression risk after adjusting demography and social determinants (model 4: OR, 1.53 [95%CI: 1.31–1.79], *p* < 0.001). We found if adjusting demography only or demography and healthy conditions, individuals with higher education levels sat more than 8 h/d is not significantly associated with increased risk of depression, suggesting some important social determinants linked with higher education level to protect them from depression. A key finding in this study was the lack of a significant association between higher education and 6–8 h of SB in relation to depression. The protective factors associated with higher education—such as better access to healthcare and more effective coping strategies—might mitigate the negative impact of SB on mental health. Future research should explore this relationship further, considering additional factors such as lifestyle, social support, and occupational stress, which may influence the effect of SB on depression in different educational groups.

Our findings underscore the dual burden borne by individuals with low educational attainment and high sedentary time, who exhibit the highest prevalence of depression and the most limited access to resources that could mitigate this risk. Our results indicate that 3.6% of participants reported less than a high school education and engaged in SB≥8 h/d (Figure 2). This phenomenon may be attributed to sedentary leisure activities, such as television viewing (TV) and smartphone usage, which belong to mentally passive SB. Previous subgroup analyses indicated that spending time watching television is correlated with a higher likelihood of depression (RR = 1.18, 95% CI 1.07–1.30), whereas computer usage was not (RR = 0.99, 95% CI 0.79–1.23) (Huang et al. 2020). Meanwhile, mentally passive SB was found to be associated with an elevated risk of depression (RR = 1.17, 95% CI 1.08–1.27), while mentally active forms of SB did not exhibit a significant impact (RR = 0.98, 95% CI 0.83–1.15) (Huang et al. 2020).

Despite the study's significant merits, such as its extensive participant pool and rigorous adjustment for confounding factors, it is crucial to recognize certain constraints. First, the cross‐sectional design precludes the possibility of establishing a causal relationship between SB, education, and depression. Longitudinal studies with repeated measures are needed to disentangle these dynamics and the underlying biological mechanisms warrant investigation in the future. Second, our analysis focused on US NHANES data, which excludes institutionalized populations (e.g., incarcerated individuals, and long‐term care residents) who face elevated depression risks and distinct SB patterns. Cultural norms around education and SB may differentially modulate these relationships, necessitating replication in diverse cohorts. Third, despite covariate adjustment, unmeasured factors could bias associations, including: (1) polygenic risk scores for depression correlate with both SB and education but were unavailable in NHANES; (2) Chronic stress from work demands, caregiving responsibilities, or financial strain—factors linked to both SB and depression—were not comprehensively captured. Fourth, self‐reported SB via GPAQ, though validated for population surveillance (Armstrong and Bull 2006), likely underestimates true sedentary time compared to accelerometry (Cleland et al. 2014). Device‐based measures (e.g., thigh‐worn inclinometers) would improve precision.

## 5 Conclusion

This study elucidates the independent and combined effects of education and SB on the risk of developing depression. Both lower education levels and prolonged SB are linked to a heightened risk of depression when considered separately and in combination. It can be reasonably deduced that interventions targeting the reduction of SB, particularly in populations with lower education levels and also in those with higher education levels who engaged in SB for more than 8 h per day, may prove effective in reducing depression prevalence.

## Author Contributions

**Chun Yang**: conceptualization, writing – original draft, writing – review and editing, investigation. **Tiankuo Gao**: writing – original draft, writing – review and editing, formal analysis. **Yichen Zhang**: writing – review and editing, software, visualization. **Cuicui Feng**: writing – review and editing, conceptualization, supervision. **Kai Zhang**: conceptualization, project administration, writing – review and editing, data curation, supervision, resources.

## Conflicts of Interest

The authors declare no conflicts of interest.

## Peer Review

The peer review history for this article is available at https://publons.com/publon/10.1002/brb3.70615.

## Supporting information




**Supporting Table 1**: brb370615‐sup‐0001‐TableS1.docx

**Supporting Table 2**: brb370615‐sup‐0002‐TableS2.docx

## Data Availability

All data are available as publicly accessible datasets through NHANES. It is open and publicly accessible through the following link: https://wwwn.cdc.gov/nchs/nhanes/index.htm.
