# Supplementary material for: Association Between Education Levels and Sedentary Behavior With Depression Among US Adults
Source: Brain Behav. 2025 Jun 10;15(6):e70615. doi: 10.1002/brb3.70615 (PMC12152267; doi:10.1002/brb3.70615)
Supplement: Supplementary file 1 — Supporting Table 1: brb370615‐sup‐0001‐TableS1.docx [file BRB3-15-e70615-s002.docx]

**Table S1. Sensitivity Analysis of Odds ratios for the association between education level and depression.**

| **Variable** | **Event/** | **Model 1** | |  | **Model 2** | |  | **Model 3** | |  | **Model 4** | |
| --- | --- | --- | --- | --- | --- | --- | --- | --- | --- | --- | --- | --- |
|  | **Participant** | **OR (95%CI)** | ***P* value** |  | **OR (95% CI)** | ***P* value** |  | **OR (95% CI)** | ***P* value** |  | **OR (95% CI)** | ***P* value** |
| **Education level** |  |  |  |  |  |  |  |  |  |  |  |  |
| Less Than High School | 989/7134 | Reference | - |  | Reference | - |  | Reference | - |  | Reference | - |
| High School Graduate | 654/6852 | 0.65(0.57,0.75) | <0.001 |  | 0.62(0.54,0.71) | <0.001 |  | 0.71(0.58,0.85) | <0.001 |  | 0.80(0.68,0.94) | 0.010 |
| Some College or AA Degree | 823/8894 | 0.61(0.52,0.71) | <0.001 |  | 0.55(0.46,0.64) | <0.001 |  | 0.70(0.58,0.85) | <0.001 |  | 0.78(0.65,0.94) | 0.010 |
| College Graduate or Above | 285/6942 | 0.25(0.20,0.31) | <0.001 |  | 0.23(0.18,0.29) | <0.001 |  | 0.44(0.34,0.56) | <0.001 |  | 0.47(0.37,0.60) | <0.001 |
| *P for trend* |  | <0.001 |  |  | <0.001 |  |  | <0.001 |  |  | <0.001 |  |

Education level was reclassified into four categories: "Less Than High School" (including "9-11th Grade" and "Less Than 9th Grade"), "High School Graduate/GED" (including "High School Grad/GED or Equivalent"), "Some College or AA Degree" (including "Some College or AA degree"), and "College Graduate or Above" (including "College Graduate or above").

Model 1: Not adjusted. Model 2: Adjusted for age, gender and sex. Model 3: Adjusted for age, gender and race, body mass index, diet score, sleep score, smoking status, alcohol consumption, hypertension, diabetes mellitus, cancer, chronic kidney disease, atherosclerotic cardiovascular disease, and educational levels. Model 4: Adjusted for age, sex, race, social determinants of health (employment status, family income-to-poverty ratio, food security, education level, regular health care access, type of health insurance, home ownership, and marital status) and educational levels.

OR, odd ratio; CI, Confidence interval.
