# Supplementary material for: Association Between Education Levels and Sedentary Behavior With Depression Among US Adults
Source: Brain Behav. 2025 Jun 10;15(6):e70615. doi: 10.1002/brb3.70615 (PMC12152267; doi:10.1002/brb3.70615)
Supplement: Supplementary file 2 — Supporting Table 2: brb370615‐sup‐0002‐TableS2.docx [file BRB3-15-e70615-s001.docx]

**Table S2. Sensitivity Analysis of Odds Ratios for the Association Between the Combination of Education Level and Sitting Time, and Depression.**

| **Variable** | **Event/** | **Model 1** | |  | **Model 2** | |  | **Model 3** | |  | **Model 4** | |
| --- | --- | --- | --- | --- | --- | --- | --- | --- | --- | --- | --- | --- |
|  | **Participant** | **OR (95%CI)** | ***P* value** |  | **OR (95% CI)** | ***P* value** |  | **OR (95% CI)** | ***P* value** |  | **OR (95% CI)** | ***P* value** |
| **High Education** |  |  |  |  |  |  |  |  |  |  |  |  |
| <6 hr/d | 464/6974 | Reference | - |  | Reference | - |  | Reference | - |  | Reference | - |
| 6 to <8 hr/d | 163/2615 | 0.98 (0.77,1.25) | 0.880 |  | 1.01(0.80,1.28) | 0.920 |  | 0.86(0.68,1.10) | 0.230 |  | 1.07(0.83,1.39) | 0.590 |
| ≥8 hr/d | 481/6247 | 1.33(1.10,1.61) | 0.003 |  | 1.37(1.13,1.65) | 0.002 |  | 1.21(0.99,1.47) | 0.070 |  | 1.64(1.34,2.00) | <0.001 |
| **Low Education** |  |  |  |  |  |  |  |  |  |  |  |  |
| <6 hr/d | 880/8467 | 1.85(1.57,2.19) | <0.001 |  | 1.95(1.64,2.33) | <0.001 |  | 1.31(1.07,1.62) | 0.010 |  | 1.36(1.13,1.63) | 0.001 |
| 6 to <8 hr/d | 259/2016 | 2.11(1.76,2.53) | <0.001 |  | 2.24(1.87,2.68) | <0.001 |  | 1.30(1.03,1.66) | 0.030 |  | 1.54(1.25,1.90) | 0.001 |
| ≥8 hr/d | 504/3503 | 2.59(2.21,3.03) | <0.001 |  | 2.74(2.33,3.22) | <0.001 |  | 1.53(1.26,1.85) | <0.001 |  | 1.91(1.59,2.30) | <0.001 |

Education level was grouped into two categories: "High Education" (including "College Graduate or above" and "Some College or AA degree") and "Low Education" (including "High School Grad/GED or Equivalent", "9-11th Grade", and "Less Than 9th Grade").

Model 1: Not adjusted. Model 2: Adjusted for age, gender and sex. Model 3: Adjusted for age, gender and race, body mass index, diet score, sleep score, smoking status, alcohol consumption, hypertension, diabetes mellitus, cancer, chronic kidney disease and atherosclerotic cardiovascular disease. Model 4: Adjusted for age, sex, race and social determinants of health (employment status, family income-to-poverty ratio, food security, education level, regular health care access, type of health insurance, home ownership, and marital status).

OR, odd ratio; CI, Confidence interval.
